# Divide and conquer: two stem cell populations in squamous epithelia, reserves and the active duty forces

**DOI:** 10.1038/s41368-019-0061-2

**Published:** 2019-08-27

**Authors:** Spencer Dunaway, Alexandra Rothaus, Yuhang Zhang, Ana Luisa Kadekaro, Thomas Andl, Claudia D. Andl

**Affiliations:** 1 Department of Dermatology, College of Medicine, University of Cincinnati, Cincinnati, OH USA; 2 Burnett School of Biomedical Sciences, University of Central Florida, Orlando, FL USA; 3 Division of Pharmaceutical Sciences, College of Pharmacy, University of Cincinnati, Cincinnati, OH USA

**Keywords:** Skin stem cells, Stem-cell niche

## Abstract

Stem cells are of great interest to the scientific community due to their potential role in regenerative and rejuvenative medicine. However, their role in the aging process and carcinogenesis remains unclear. Because DNA replication in stem cells may contribute to the background mutation rate and thereby to cancer, reducing proliferation and establishing a relatively quiescent stem cell compartment has been hypothesized to limit DNA replication-associated mutagenesis. On the other hand, as the main function of stem cells is to provide daughter cells to build and maintain tissues, the idea of a quiescent stem cell compartment appears counterintuitive. Intriguing observations in mice have led to the idea of separated stem cell compartments that consist of cells with different proliferative activity. Some epithelia of short-lived rodents appear to lack quiescent stem cells. Comparing stem cells of different species and different organs (comparative stem cell biology) may allow us to elucidate the evolutionary pressures such as the balance between cancer and longevity that govern stem cell biology (evolutionary stem cell biology). The oral mucosa and its stem cells are an exciting model system to explore the characteristics of quiescent stem cells that have eluded biologists for decades.

## Introduction

Adult stem cell biology has flourished in recent years, and one hotly debated topic in the field is the rate of proliferation of stem cells. There is evidence that several epithelia may contain two stem cell compartments; one that is slow-cycling (referred to as quiescent) and another that is proliferative, acting as the main driver of tissue maintenance (referred to as active).^1^ Whether there is a true cellular separation between quiescent and active stem cells and what the purpose of this compartmentalization is, remains unclear and controversial. This review seeks to revisit the evidence, significance and potential role of quiescent stem cell populations for tissue homeostasis and longevity of mammals, with a focus on human squamous epithelia, in particular the oral mucosa. The squamous epithelia lining the oral cavity and the esophagus exhibit unique features in regard to its basal cell layer, which make these epithelia prototypic examples harboring quiescent stem cell populations. These unique features of human oral mucosa can be exploited to better understand what the key characteristics of quiescent stem cells are in regard to DNA damage control, protein translation characteristics, metabolism and epigenetics, and how these characteristics may have been modulated by evolution to adapt to the needs of long-lived (humans) and short-lived (mice) animals.

## Peto’s paradox and stem cell quiescence

Before discussing the evidence for dual compartment stem cell populations, it is important to understand what purpose quiescent stem cells might play. Peto’s paradox describes the discrepancy between the number of cells that can function as cancer-initiating cells in small and large mammals and the actual rates of carcinogenesis.^2^ That means that a large animal has substantially more cells than a small animal and therefore the likelihood of malignant transformation should increase with body size/body cell number. Peto’s paradox integrates the importance of time into the relationship between body mass, longevity and cancer rate. The paradox that most large mammals being long-lived and most small mammals being short-lived has been recognized already by Aristotle,^3^ explored by Max Rubner^4^ and critically reviewed by Speakman.^5^ It has been estimated that the cancer risk for large/long-lived mammals should be a trillion times higher than the risk for a mouse.^6^ However, in fact both mice and humans may have similar incidences of cancer by the end of their lifespan (~30%), this is despite the over 100 year age gap between the species.^7^ Some aging studies in mice show even higher cancer rates affecting up to 90% of the animals and suggesting the possibility that laboratory mice are prone to cancer.^8^ Similarly, despite having thousands of times more cells and stem cells, of which many may be potentially cancer-initiating, whales and elephants succumb to cancer at lower frequencies than humans.^9,10^ Several evolutionary mechanisms by which large/long-lived mammals evade increased cancer risk have been offered to explain Peto’s paradox,^6,11,12^ one of which involves quiescent stem cells and the existence of dual stem cell compartments.

Unfortunately, we know very little about stem cells in large mammals, even human stem cell compartments are surprisingly poorly characterized.^13^ However, it is a widely held belief, although not undisputed,^14–16^ that the rate of stem cell proliferation is linked to malignant transformation of stem cells into cancer cells or cancer-initiating cells. It is also generally believed that the origin of cancer lies within the stem cell compartment and that the mutations within the stem cell compartment can lead to cancer, especially in conjunction with external factors such as wounding or inflammation.^17^ On the basis of these ideas, lowering the rate of stem cell proliferation, thus rendering them quiescent, should reduce cancer rates and enhance longevity. Indeed, mathematical models have provided support for this idea.^16^ This leads to the simple hypothesis that long-lived mammals may exhibit a larger quiescent stem cell compartment in many of their tissues compared to short-lived animals. Comparative stem cell biology has already established that stem cell compartments seem to increase with longevity. For example, in mice, the size of the hematopoietic stem cell pool varies by strain and correlates with their lifespan. Animals from longer lived strains have bigger stem cell pools.^18^ However, it remains unclear whether the increased stem cell pool encompasses the entire stem cell population or only a quiescent component, and whether this is proportional to the size of the animal. This concept can also be applied at the tissue level. If lifetime stem cell proliferation events are linked to cancer, then tissues with high turnover or high proliferation rates would be disproportionately exposed to replication-associated cancer risk. Tissues such as the squamous epithelia, intestinal epithelia and the hematopoietic system would require a solution to reduce replication errors in their stem cells, potentially by increasing the proportion of quiescent cells. Engagement of the scientific community in comparative stem cell biology combined with the collection, preservation and analysis of tissue samples, especially of large/long-lived mammals, may reveal important insights into how different animals maintain their tissues in response to different requirements to deal with longevity and prevent tumorigenesis.

## Human squamous epithelia: breaking the dogma of the localization of the quiescent niche

**Fig. 1:**
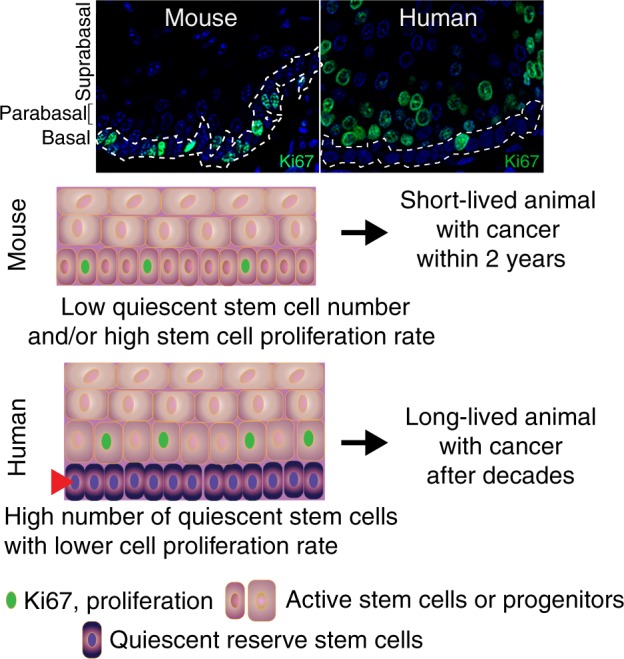
Organizational shift in squamous epithelia of small short-lived (mouse) to long-lived mammals (human): the establishment of a quiescent basal cell layer overlaid by a proliferative parabasal cell layer

**Fig. 2:**
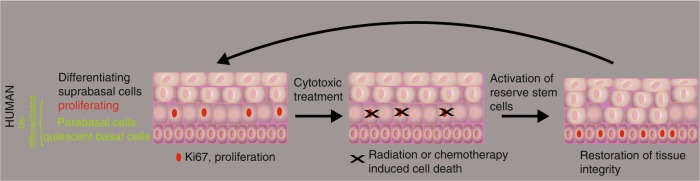
Model how catastrophic events such as cytotoxic treatments trigger the activation of the quiescent stem cell layer similar to observations made in mouse colon and the HSC system

Quiescence is often defined by the absence of proliferation marker expression. The dogma for squamous epithelia, including the outer root sheath of the hair follicle and its bulge region, is that stem cell populations can be found in the basal cell layer and that proliferation within the epithelium is essentially restricted to this basal cell layer. This means that stem cells, quiescent and active, and transient-amplifying cells (TA cells) intermingle in the basal cell layer (Fig. 1). This intermingling of active and quiescent stem cells as well as TA cells within the basal cell layer may be one of the reasons accounting for the difficulties in determining the nature of the squamous epithelial stem cells in the mouse. However, for about 50 years it has been well-established that proliferation in human squamous epithelia, such as the cervix and vagina, takes mainly place suprabasally or in the first suprabasal cell layer, the parabasal cell layer, whereas proliferation is rare in the basal cell layer.^19,20^ This deviation of the basal cell proliferation dogma is not only true for ectocervix and vagina, but also for the oral cavity, the esophagus and the anus (Fig. 1^21^). Even in the human epidermis the situation is not as clear cut as the dogma states, and a substantial number of replicating cells can actually be found in suprabasal cell layers^21,22^ and has been debated for nearly 100 years.^23–25^ In the human oral mucosa, these proliferating and undifferentiated non-basal cells are frequently associated with the parabasal cell layer, and one interpretation of this organization is that the stem cell compartment is divided in a basal quiescent and a parabasal active compartment similar to what has been outlined for the stem cell compartments of the intestines, the hair follicle and the hematopoietic system. In addition, similar to the response of intestinal and hematopoietic stem cells, “catastrophic events” that kill many cells within the epithelium such as lethal radiation seems to eliminate the putative active stem cell compartment in the parabasal cells and activates the quiescent basal stem cells of the human oral mucosa. This has been observed in human tissue samples from patients during and after radio-chemotherapy (first report: ref. ^26^, Fig. 2). Human oral mucositis, the response of the oral mucosa towards radio-chemotherapy, is characterized by the elimination of proliferating parabasal cells and a shift of proliferation to the previously quiescent basal cell layer (Fig. 2).

## The limitations of using label-retaining cell analysis to identify quiescent self-renewing cells in human squamous epithelia

Recent studies by Jankowski’s group^27^ using human tissue samples have lent support to the idea of a quiescent reserve stem cell layer in the esophagus: label-retaining cells (LRCs) are restricted to the basal cell layer in the human esophagus. This may not sound surprising but keep in mind label-retaining experiments using BrdU or similar reagents have two major limitations. First, they can only detect cells that actual take up enough “label”, i.e., BrdU during the labeling period. This labeling period has to be relatively short in humans due to the fact that these toxic drugs cannot be perfused for prolonged periods. Their toxicity is the second limitation; it is unclear how cells will behave and react during another round of DNA replication if many nucleotides have been substituted with BrdU. The first limitation is serious as basal cells are quiescent and only a few percent of basal cells are Ki67-positive.^21,22,28^ If few cells are going through S-phase during the labeling period, few cells can incorporate the label, and few of these normally quiescent stem cells will be detected subsequently. In the human squamous epithelium of the cervix, it has been estimated that a basal stem cell replicates every 33 days,^20^ and therefore a labeling period of at least 33 days would be necessary. On the other hand, BrdU label should easily be incorporated into the DNA of many parabasal cells of human squamous epithelia as they compromise the bulk of proliferating cells and divide in the ectocervix about every 3 days.^20^ Therefore, the fact that only basal cells retain label in the experiments conducted by Pan et al.^27^ is extraordinarily informative. All the long-term label-retaining cells, in their case the label has been IdU (5-iodo-2′-deoxyuridine), is restricted to the basal cell layer. The surprising part is that despite most of the labeling initially occurring in the parabasal cell layer, label-retaining cells are only found in the basal cell layer with the longest chase period in the patient cohort being 67 days. These findings indicate that the basal cells are overall quiescent and long-lived.

If we now take into account the basic biophysical properties of IdU, i.e., a circulation half-life of 8 h, and an infusion time of 30 min, then only a tiny fraction of basal stem cells can be labeled. Sufficient uptake of IdU was most likely restricted to a day or less due to infusion time and half-life of IdU, which means that on average not >3% of basal cells should accumulate enough IdU (as mentioned above, the average cycle time of a basal cell in the cervix is 33 days). The number of labeled basal cells detected after seven days by Pan et al. is indeed exactly as predicted: 2.3% of basal cells were IdU positive. These results are in striking contrast to the findings in mouse esophagus (reviewed by Jones and Klein^13^) where LRCs seem to be absent or when present, do not constitute keratinocytes.

## The control of quiescence and G0 in stem cells

**Fig. 3:**
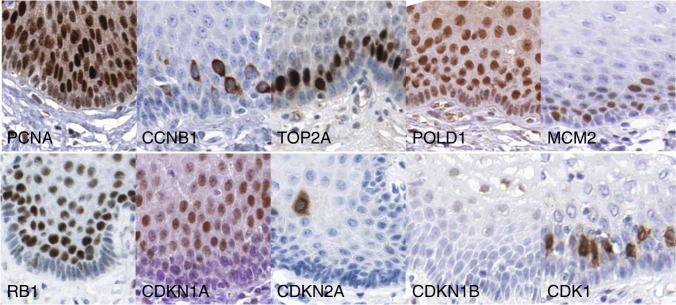
Expression of cell cycle regulators, proliferation markers and CDK inhibitors in human squamous epithelia. Images courtesy of The Human Protein Atlas available from www.proteinatlas.org

Quiescence is a frequently used term in stem cell biology and is tightly linked with the term G0 phase of the cell cycle^29^ indicating that the cells have left the cell cycle. Quiescence can represent itself on the transcriptional level in several forms and variations indicating that the quiescent state by itself is complex.^30,31^ Moreover, the vast majority of molecular studies of cellular quiescence have been undertaken in vitro. Overall, the G0 phase is poorly defined at the molecular level. Its occurrence is often described in conjunction with truly post-mitotic cells such as neurons and differentiated cells such as hepatocytes. Recent advances in G0 biology may help, but it remains to be seen whether the type of quiescence observed in vitro and the markers associated with in vitro quiescence faithfully reflect the situation in vivo.^32^ However, it can be agreed on that the lack of expression of basically all classical cell cycle markers (for example, cyclins, CDKs, RB1) defines indeed a form of G0 state (Fig. 3).

Our analysis of human oral tissues identified the lack of proliferation markers including Ki67, PCNA and certain cyclins in the basal cells.^21^ In addition, almost every cell cycle-associated marker is absent in oral mucosa basal cells but expressed in proliferating parabasal cells. This indicates that the basal cells may have indeed completely withdrawn from the cell cycle and progressed into “deep” quiescence/G0. Therefore, it seems likely that the quiescent basal cell in the human oral mucosa represents a novel promising model to define what it means to be quiescent and what G0 entails in vivo. Often G0 and quiescence are associated with differentiated cells. However, quiescent stem cells are clearly undifferentiated and ostensibly divide to provide differentiated progeny. Therefore, these stem cells may have different G0 characteristics than truly post-mitotic cells, including differentiated and senescent cells. It will be interesting to elucidate how this stem cell G0 state is overcome and how they enter the G1 phase, a capability not seen by most cells in the body.

## The DREAM complex and quiescence and its relevance for G0

**Fig. 4:**
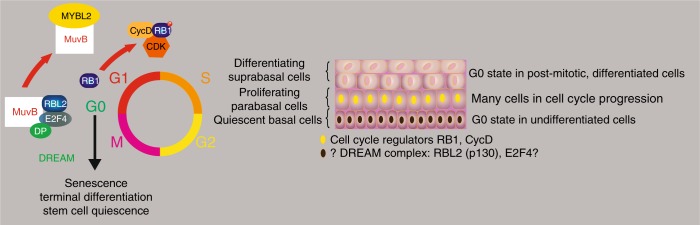
The potential role of DREAM complex components in oral stem cell quiescence. On the basis of the data on the DREAM complex, the hypothesis can be stated that the core components of the G0-maintaining and -inducing DREAM complex, such as RBL2 and E2F4 are expressed in the quiescent basal cell layer of the oral mucosa. However, currently there are no data supporting this hypothesis. However, RB1 and the machinery that phosphorylates RB1 are present in para- and suprabasal cells but absent in the basal cells (see also Fig. 3). On the other hand, MYBL2 may be expressed broadly in the squamous epithelium of the esophagus but with a tendency to higher expression in basal cells, an expression pattern that does not fit the proliferative state of the cells^155^

One of the more prominent G0/early G1-associated molecular signatures involves the DREAM complex (Fig. 4). The DREAM (DP, RB1-like, E2F and MuvB) complex is an important suppressor of core cell cycle regulator promoters.^33^ The specific factors that may mediate its G0 activity are E2F4/5 and RB1-like factors RBL1 and RBL2, also known as p107 and p130. One could speculate that the quiescence and depth of quiescence of stem cells therefore is mediated by DREAM activity.^31^ The situation is complicated by a heavy overrepresentation of DREAM target genes involved in the S-phase of the cell cycle. For example, it includes many histones that are generally believed to be produced in late G1, early S-phase. This may seem to be counterintuitive for a true G0-maintaining protein complex and may indicate that the DREAM complex is rather a G1/S regulator. However, it is difficult to distinguish between a G1/S and a G0/S transition. This disadvantage of the current DREAM dataset may be due to its reliance on in vitro data. Although the activity of the DREAM complex is an attractive and high-profile case in quiescence biology, it does not seem to fully explain stem cell quiescence in vivo. The overrepresentation of late G1 or S-phase genes regulated by this complex(es) may not fit the requirements of a control element mediating the transition from proliferation to quiescence and more importantly to maintain G0. However, at the moment the DREAM complex is one of the few clues we have to explain quiescence at the molecular level. But so far, little evidence has emerged that implicates DREAM in the biology of quiescent stem cells. An interesting starting point could be the analysis of the expression of key DREAM components E2F4/5 and RBL1/2. To our knowledge neither the literature nor data collections, such as the Human Protein Atlas (HPA), provide evidence that these proteins are expressed in the quiescent basal cell layer of human squamous epithelia or any other quiescent stem cell population.

An elegant study addressing G0 in vitro^32^ showed limited overlap between the genes identified in G0 cells and markers expressed in quiescent basal cells of human squamous epithelia. Oki et al.^32^ also described enrichment of certain epigenetic regulators in quiescent fibroblasts. However, they found that many cell cycle regulators such as RB1, CDK inhibitors or p53 were elevated in quiescent cells in vitro. None of these markers seem to be expressed in the quiescent basal cell layer of the oral mucosa (Fig. 3) suggesting that different forms and levels of quiescence may exist and that quiescence can come in different flavors in vitro and in vivo.^30,31,34^ Therefore, it is unclear whether quiescence in vitro and in vivo mean the same thing at the gene expression level. The lack of a set of well-defined molecular biomarkers for quiescence/G0 has hindered translation of in vitro quiescence data into in vivo. For example, proposed markers such as RB1 (ref. ^35^) and the CDK inhibitors p27 (CDKN1B) and p21 (CDKN1A)^36^ are not expressed in quiescent basal cells of squamous epithelia (Fig. 3).

A critical factor that may explain the differences between in vitro and in vivo quiescence may be the lack of a specialized niche in vitro, an environment that may keep the stem cells quiescent (see the comments in ref. ^37^). Accounting for physiological context, architecture and microenvironment, analysis of quiescence in organoids or spheroids may better promote our understanding of quiescence than studies performed in 2-dimensional cultures.

Other mechanisms that have roles in regulating stem cell quiescence and G0 could be secreted factors. Interestingly, many genes expressed in the basal cell layer have been implicated in TGFbeta signaling or regulation by TGFbeta signaling.^21^ This gives a clue to not only how quiescence may be induced but also maintained in this cell layer. TGFbeta signaling is able to inhibit stem cell proliferation and is well known to induce cell cycle arrest in keratinocytes.^38–40^ Overcoming this inhibition has also been shown to contribute to squamous epithelial carcinogenesis.^39,41^ However, due to the unique organization of human squamous epithelia, no real experimental system is available to further test this idea and determine how TGFbeta signaling exactly regulates squamous epithelial stem cell biology in humans. In mouse models with impaired TGFbeta signaling in squamous epithelia, we observed two contradictory events: the loss of Tgfbr2 caused epithelial atrophy and eventually squamous cell carcinoma (SCC) formation. SCCs may form because TGFbeta is a major inhibitor of keratinocyte proliferation and its loss takes away one roadblock to tumorigenesis.^42^ However, atrophy may be attributed to stem cell exhaustion after activation of quiescent stem cells and the selection of clones that can overcome this atrophy, eventually leading to SCCs. In vitro, manipulation of TGFbeta levels can induce features that reflect their status in the human body in the basal cell layer of an artificial mucosa using human oral keratinocytes 3D organotypic cultures. However, even 3D artificial tissues cannot recapitulate the key structural element of the human epithelium: in these artificial mucosae, proliferation takes place in the basal cell layer. However, the problem seems to lay in the lack of fine-tuning the TGFbeta signal and the usage of cultured and therefore “activated” wound-like keratinocytes, that might be harder to tame than naïve keratinocytes in vivo. It is interesting that TGFbeta signaling can reduce the effects of cytotoxic treatments in mouse epithelia likely through reducing the proliferation of active stem cells.^43^ In conclusion, there is mounting evidence that TGFbeta signaling has a key role in quiescent stem cell biology and a proper balance between its activation and inhibition is required to maintain tissue homeostasis.

## Quiescence stem cells are at the heart of mutation susceptibility and life expectancy

Protecting the stem cell compartment from DNA damage is not an easy task for a long-lived animal and requires considerable effort. How difficult this task is may be illuminated when considering the endogenous somatic mutation rate in human cells in vivo. Adult human cells, e.g., skin fibroblasts have accumulated hundreds of mutations even in sun-protected areas most likely due to replication errors^44,45^ suggesting that aging humans gradually harbor mutations in cancer-driver genes and that exogenous factors such as ultraviolet (UV) light further predisposes the rate of mutation to a level that frequently results in cancer. In sun-exposed human epidermis, the burden of somatic mutations averaged two to six mutations per megabase per cell.^46^ From studies with human organoid cultures derived from stem cells from donors of ages varying from 3 to 87 years-old, it has been shown that the mutational load increases steadily over time at a rate of approximately 40 novel mutations every year.^47^ Although it is unclear whether this rate of mutations is sufficient to drive cancer development in different tissues, it highlights the existence of mechanisms that protect stem cell populations and delay the accumulation of cancer-driver mutations until reaching the end of the reproductive age. Reduction of mutation rates can be achieved by attenuating endogenous and exogenous deleterious effects on DNA integrity. The observation that human somatic cells “accumulate mutations 4 to 25 times more rapidly than germline cells do”^48^ suggests the existence of potential areas of intervention by which the mutation burden load could be further reduced. Establishment of a “deep quiescence” state^49^ in the stem cell population, along with enhanced detoxification and more efficient DNA repair may keep the mutational load low enough to prevent cancer beyond the reproductive age and enhance the natural life expectancy.

A relationship between life expectancy and mutation rate has been suggested by Failla in the 1950s, when he stated that “the important point is that the average spontaneous mutation rate of a somatic cell of a short-lived species be higher than that of the somatic cells of a long-lived species and approximately in the inverse ratio of the life spans”.^50^ Whether Failla has been right is still unclear but a recent study by Blokzijl et al.^47^ summarized some of their findings by saying that “although variation in tissue-specific mutation spectra in mice has been reported previously, we observed a difference in both mutation rate and spectrum in human cells. This indicates that mutation data derived from mice are not necessarily suitable for interpreting mutational processes and their consequences in humans.”^47^ Similarly, Vijg et al.^51^ suggest that mutation rate per base and replication in mouse germ cells and somatic cells is significantly higher than in humans. In their Fig. 6, they state: “The somatic mutation rate was nearly two orders of magnitude higher than the germline mutation rate in both species; in mice, both the germline and somatic mutation rates were several times higher than their human equivalents.” This may explain why in some studies the cancer rate in mice is considerably higher than in humans.^8^

One of the studies on somatic mutational load in human fibroblasts suggested that the majority of these mutations occur during development when the proliferation rate is high, whereas few are added in adult life when proliferation in fibroblasts is low.^45^ This supports the notion that replication is a “dangerous” process associated with errors that can accumulate astonishing numbers of mutations over time and form the platform on which cancer cells may build upon. E.g., human cells of self-renewing tissues such as colon and intestine add about 40 mutations every year.^47^

There is growing evidence that understanding human adult stem cell biology and the factors regulating stem cell proliferation and quiescence will help to improve our ideas about the carcinogenic process as well as conditions associated with aging and tissue degeneration. The complexities of stem cell biology and human stem cell terminology and nomenclature are still confusing.^52^ Although quiescent stem cells in the mouse can rescue tissues after catastrophic irradiation, the question remains whether this response to massive cell death is the true purpose of such quiescent stem cells. It is interesting to note that these putative reserve stem cells, activated by catastrophic events, are often found in the digestive tract (intestine crypts, basal cell layer of oral, esophageal and anal squamous epithelia). Quiescence of these cells may also guarantee that even if cytotoxic reagents taken up with food, these toxins will not destroy the entire stem cell compartment but just the “lower” quality active stem cell compartment. Recently, a similar idea has been tested in intestinal crypts.^53^ Kaiko et al.^53^ claim that metabolites from the microbiome can have inhibitory effects on crypt stem cells but the structural organization of the crypt prevents that such metabolites reach critical concentration at the base of the crypt and therefore will generally not affect the stem cells. The principal idea is that the stem cell compartment may require a protective shield and a back-up system that prevents toxic products passing through or generated within the digestive system from causing irreversible damage. The ability to overcome this threat by having a relatively inert reserve stem cell population that can repair the tissue once the active stem cell population is exhausted, could be advantageous. The catastrophic events that are brought upon experimental mice or human patients undergoing radio- and chemotherapy may only represent extreme situations, in which the reserve stem cell compartment becomes evident. In general, these reserve stem cells may work undetected and may have evolved to confront the acute problem of toxic substances impairing tissue and stem cell function.

Another question is whether quiescent stem cells are actually required for a short-lived animal like the mouse. As Clevers pointed out “it appears somewhat counterintuitive that cells whose only raison d'être is the generation of daughter cells, would rarely divide”.^52^ From an evolutionary point of view, does a mouse need quiescent stem cells to achieve the major function quiescent stem cells supposedly have: to maintain a tissue over prolonged time with minimal stem cell divisions to avoid tumorigenesis and repair the tissue once the active stem cells have been eliminated? Even if this hypothesis is true for humans, little evidence substantiates the idea that relative quiescence reduces tumorigenesis in an animal with a lifespan of several decades. Many unanswered questions await a new generation of comparative and evolutionary stem cell biologists. However, if quiescence reduces mutation rates and thereby enhances organismal survival then accumulation of mutations and mutation in genes regulating DNA repair should at least be associated with aging and tumorigenesis (somatic mutation theory combined with a stem cell theory of aging). Indeed, basically all known premature aging syndromes have a connection to DNA repair or nuclear architecture. Furthermore, mutations accumulate with age, DNA repair systems deteriorate with age and many models of impaired DNA repair result in reduced lifespan (summarized in ref. ^54^). Both these theories of aging (mutational and stem cell) can be closely related especially if adult tissue stem cells can become cancer-initiating cells and accumulation of mutations in stem cells leads to senescence or impaired stem cell function. There is some evidence that defects in stem cell DNA repair contribute to aging.^55–58^ Whether short-lived mammals have lower quality DNA repair systems than long-lived mammals is unclear and may not be necessary when focusing on stem cells and their protection by quiescence rather than by higher DNA repair rates.^59^ Again, our descriptive findings in human oral mucosa indicate that the quiescent basal cell layer expresses elevated levels of several key repair genes (e.g., XPC and associated factors).^21^ It is interesting that several experiments on stem cell populations and especially quiescence have identified an innate immune system association.^32,60^ Oki et al.^32^ report that the top six gene ontology (GO) terms associated with quiescent fibroblasts are all related to inflammation, immune defense and wound response. Protecting the quiescence stem cells on every level that may alter its genetic integrity (DNA mutations and integration of viral genomes, e.g., HPV) seems to be the priority of the quiescent state. Therefore, gene signatures relevant for DNA repair and innate immunity may be characteristic for quiescent stem cells.

## Quiescent cells display metabolic activity

Since our theoretical framework requires stem cells to be in mint condition for many years in mammals with a high maximum age limit, we assume that factors diminishing the fitness of stem cells will be limited at least during the reproductive age of an organism. What immediately comes to mind is oxidative stress stemming from mitochondrial oxidation. Although this process is energy-efficient, it produces potentially harmful reactive oxygen species (ROS). This risk can be reduced using alternative, but less productive pathways such as glycolysis. Frequently, glycolysis is increased in a hypoxic environment.^61^ Interestingly, the niche of some stem cells has been determined to be hypoxic.^62^ Combined with the fact that many stem cells are quiescent, it seems an easy decision to postulate that stem cells avoid sources of “unnecessary” ROS production by relying on less “polluting” processes to cover their limited energy needs. There is evidence that reduced metabolic activity may be a stem cell characteristic.^63^ However, insights into this issue are limited to a few types of adult stem cells and have not been extended to self-renewing epithelial tissues such as the oral mucosa. The evidence for a hypoxic niche for squamous epithelia with utilizing primarily glycolysis is slim. The fact that cells do not proliferate does not mean they do not need ATP. The brain, for example, requires a substantial amount of power, disproportionate to its size and proliferation rate. This is most likely due to ATP-intensive processes such as maintaining gradients and “pumping” molecules across membranes. Although it is not known whether and how basal cells establish certain gradients and whether they function in part as feeder cells for the overlying epithelium, there is good evidence that the activity of key Krebs cycle enzymes indicative for high energy product is as elevated in basal cells as in the proliferating parabasal cells in vivo.^64–66^ The data from Mori’s group even suggest specific or at least predominant “succinic dehydrogenase activity” (SDH) reflecting the expression of the mitochondrial succinate dehydrogenase enzyme complex in the basal cell layer of human oral mucosa.^67^ This is supported by later studies on the mRNA expression of mitochondrial genes in human esophagus,^68^ by protein expression of SDH protein (HPA), and ultrastructural analyses of human oral epithelia, which found that the quiescent basal cell layer appears to have the highest amount of mitochondria, often concentrated on their basal side, compared to suprabasal and differentiated keratinocytes.^69,70^ All these data collectively support the idea of a metabolically active basal cell layer despite its proliferative inactivity. Even in vitro there is now evidence that quiescence may actually be associated with a high metabolic rate.^71^

It is plausible to postulate that the quiescent stem cells harbor a more elaborate machinery to deal with oxidative stress. Our analysis of genes expressed in human squamous epithelial basal cells indicate that they express a protective signature.^21^ In fact, there is evidence that transplanted stem cells exerts antioxidant effects in liver^72^ as well as in diabetic mice.^73^

## Translational control of proteostasis in stem cells

It is a well-accepted notion that the quality and efficacy of DNA replication and DNA repair machinery correlates with longevity. An animal with a long lifespan should have an excellent DNA repair system and make few mistakes during replication. Thus, ensuring that critical errors do not accumulate to an unsustainable level during the reproductive age and thereby diminish fitness. But how about translational control, translational activity, the error rate of translation and protein folding and elimination of faulty proteins in relationship to longevity? Does the quality of the proteome of adult stem cells affect longevity?^74^ The quality of the proteome of stem cells and for that purpose of all cells rest on several pillars: translation accuracy, efficient folding, appropriate protein production rate and elimination of damaged proteins. All these pillars are important for proteome homeostasis or proteostasis. Deficiencies or imbalances in any of these pillars affect adult stem cell function and are associated with, correlate with or cause aging, disease and cancer.

First, there seems to be little evidence that translational fidelity decreases with age or drives the aging process per se (summarized in ref. ^75^). However, mutations affecting fidelity of protein translation can have severe effects and reduce lifespan.^76^ Few studies have started to elucidate whether the accuracy of protein translation correlates with longevity. In rodents, this indeed seems to be the case and there are dramatic differences, e.g., between mice (short-lived) and the naked mole rat (long-lived): the naked mole rat has ten times better translation fidelity than the mouse.^75,77^ Some evidence that can be interpreted as a role of translational accuracy in stem cell biology comes from Trdmt1/Dnmt2 KO mice. Dnmt2 methylates tRNAs at a specific site which protects the tRNA from degradation and enhances the accuracy of codon recognition by the methylated tRNAs.^78^ The experiments by Tuorto et al. show that Trdmt1 (Dnmt2) loss alters the proliferative capacity of hematopoietic cells and alters their differentiation. Another cytosine-5 tRNA methylase, NSun2, has been implicated in regulating stem cell function.^79^ In contrast to Trdmt1 (Dnmt2) whose loss increases proliferation, overexpression of NSun2 enhances proliferation. Accordingly, suppression of NSun2 in keratinocytes reduces their proliferation.^80^ Beyond proliferation, NSun2 also seems to affect stem cells: loss in mice has been associated with a hair follicle stem cell renewal deficit.^81^ In the mouse hair follicle, NSun2 expression was associated with some of the most highly proliferative cells in the body, the hair follicle matrix cells. NSun2 is mainly expressed in suprabasal cells in mouse and human epidermis and parabasal and suprabasal cells in human esophagus and oral mucosa. This means that NSun2 expression levels are low or absent in the stem cells of these squamous epithelia. This is in line with the findings of Blanco et al.^81^ from Frye’s laboratory that quiescent undifferentiated cells have low NSun2 levels. In a follow up study, Dr. Frye’s group then showed that keratinocyte stem cells have lower protein synthesis levels than their committed progeny partly due to low NSun2 levels.^79^ They also suggested that more aggressive human SCCs are associated with lower NSun2 levels, which mediates lower translation rates and the expansion of a more stem cell-like state.^79^

A protein inhibitor of the tRNA hydrolyzing enzyme angiogenin is RNH1, and RNH1 is co-expressed with NSun2 in human squamous epithelia in non-stem cells (see HPA^82–84^) suggesting that the protection of tRNA integrity by their cytosine-5 methylation (NSun2) and inhibition of their cleavage by angiogenin (RNH1 and methylation by NSun2) allows non-stem cells to have high levels of protein translation while stem cells are relatively “quiescent” in this regard, too.

Expression of tRNAs themselves may be a major regulator of the quiescent, proliferative and differentiated states.^85^ Although the mechanism of coordinating a specific subset of tRNAs to optimize codon usage with proliferation and differentiation has not been applied to stem cell biology specifically, the work of Gingold et al. strongly suggests that quiescent stem cells have a distinct tRNA pool and expression pattern that reflects their translational activity.

Another crucial aspect of proteostasis is correct protein folding and refolding of unfolded proteins. During aging this facet of proteome health seems to deteriorate.^86^ However, little is known about the role of protein folding in adult epithelial stem cells and little data support the role of chaperones in hematopoietic and neural stem cells.^87^ We performed a survey of the expression of most members of the major chaperone families, which govern protein folding using the Human Protein Atlas^84^. At least five members of the HSP70 family are preferentially expressed in the basal cell layer of human squamous epithelia according to HPA data including HSPA1A, HSPA1B, HSPA8, HSPA6 and HSPA2 (Supplementary Figure S1). In the epidermis and keratinocytes, HSPA2 is required to prevent premature differentiation and maintain clonogenicity^88^ implying an important role of this chaperone in maintaining an undifferentiated, stem cell-like state of keratinocytes. Interestingly, the HSPA2 co-chaperone DNAJB14 from the HSP40 family is also preferentially expressed in the basal cell layer of human squamous epithelia similar to other factors directly interacting with HSPA2 (according to HPA). These other factors relevant for chaperone activity include, e.g., BAG3. Collectively, these expression data imply a constitutive presence of a HSP70 family complex in the quiescent stem cells of human squamous epithelia, and that this complex may maintain an undifferentiated and quiescent state of these cells.^88^ Otherwise, there is very little known about how chaperones regulate adult stem cell behavior and stem cell proteostasis. Much deeper is our understanding of the regulation of the protein translation rate and its impact on stem cells. A general rule is that reduced protein translation rates correlate with longevity. In *C. elegans* and *Drosophila*, for example, the inhibition of protein translation extends lifespan.^89^ One of the now classical life extending treatments is the inhibition of the mTOR signaling pathway that mediates control over protein translation rates.^90,91^ In mouse skin, rapamycin, a mTOR inhibitor, can reverse the effects of Wnt1-mediated hair follicle stem cell exhaustion.^92^ The Gutkind laboratory also could show a beneficial effect of rapamycin on the clonogenicity and proliferation of human oral keratinocytes and a protective function in mice against oral mucositis induced by radiation treatment.^93^ Rapamycin also dramatically prolonged the lifespan of primary keratinocyte cultures, likely by suppressing keratinocyte senescence. These results are astonishing if one considers the major side effects of rapamycin treatment on the human oral mucosa in organ transplant or cancer patients. Rapamycin can cause so called mTOR inhibitor-associated stomatitis, which seems to be triggered mainly by reduced proliferation and death of keratinocytes in response to rapamycin. This initiates the development of ulcers, which can paradoxically be treated with another class of immunosuppressive drugs, corticosteroids. It is difficult to reconcile the findings of the Gutkind laboratory and the real-world experiences of patients with painful oral lesions while on rapamycin. Also, in 3d models of oral mucosa, rapamycin had a profoundly negative impact on keratinocyte proliferation and health.^94^ Furthermore, activation of the mTOR signaling pathway by knocking out one of its negative regulators, Tsc1, in hematopoietic stem cells abolishes stem cell quiescence.^95^ Therefore, in general, inhibition of mTOR signaling seems anti-proliferative. How mTOR inhibition in human keratinocytes in vitro can have profoundly positive effects on their health, proliferation potential and clonogenicity^92^ may depend on the fact that the cells are in an activated state in vitro, while the stem cells in vivo in human oral mucosa—or in the hematopoietic stem cell system—are in a quiescent state. This line of thought fits the idea that mTOR signaling favors senescence, which is quickly attained when cultivating keratinocytes in vitro. Therefore, in vitro, rapamycin’s major effect on keratinocytes may be the suppression of senescence as has been shown by the Gutkind group.^92^ Whether rapamycin really can inhibit senescence and proliferation in squamous epithelial cells in a context dependent manner is still unclear. Here, a remarkable case study may be of interest in which rapamycin reduced skin cancer rates compared to other immunosuppressive drugs in a heart transplant patient but also dramatically slowed down wound healing. Upon rapamycin withdrawal and replacement with other immunosuppressive reagents, wound healing was restored but also skin carcinogenesis accelerated again.^96^ All these human in vivo data suggest that rapamycin inhibits keratinocyte growth.

On the other hand, the data from the Gutkind laboratory could be interpreted as support of the idea that quiescence is a powerful stem cell protective mechanism. Rapamycin may in vivo reduce the proliferation rate and thereby protect the transient-amplifying cells (TA) cells, active stem cells and active progenitor cells from the deleterious effects, e.g., of radiation.^93,97^ This bears the question: where is mTOR mainly active in squamous epithelia? Most likely in differentiated cells that seem to be the protein factories of squamous epithelia and express almost exclusively the classical markers of active mTOR signaling such as pRPS6 (also known as pS6) or pEIF4EBP1 (better known as p4EBP1).^79,98–103^ Indeed, loss of mTOR in mouse epidermis exactly produces this phenotype: loss of barrier function due to abnormal keratinocyte differentiation.^100^

The data on the mTOR effects on the stem cell compartments of different lineages and tissues support the notion that mTOR promotes proliferation and differentiation although the picture is still blurry and littered with opposing verdicts.^104^ In keratinocytes and hair follicle stem cells, the data are relatively clear and mTOR signaling activates proliferation, i.e., promotes the activation of quiescent stem cells, e.g., by inhibiting the growth suppressive action of BMP signaling.^105^ Fitting to this line of argumentation is the fact that DEPTOR, a mTOR inhibitor, is mainly expressed in the quiescent basal cells of the human esophagus^106^, which is confirmed by data from the HPA (see also Supplementary Figure S2).

**Fig. 5:**
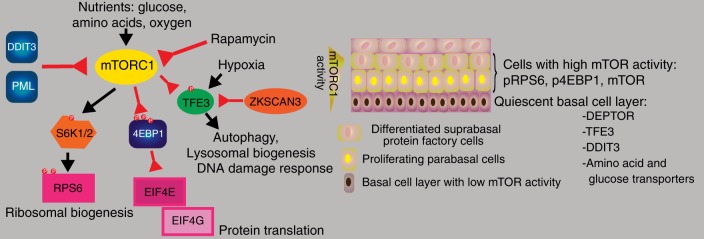
Model of mTOR signaling in the oral mucosa based on expression data of key targets of mTOR activity (S6K1/2, RPS6, EIF4B, TFE3, EIF4E, EIF4G) and putative regulators of mTOR (DEPTOR, PML, DDIT3). Therefore, the cell layers with the highest mTOR activity may be parabasal and especially suprabasal differentiated cells. Some expression data for these proteins are summarized in the Supplementary Fig. S2

As mentioned before, the core targets of mTOR are S6K1/2 (RPS6KB1 and 2) and EIF4EBP1. In addition, TFE3 and ULK1 are inhibited by mTOR signaling.^107^ TFE3 regulates a surprisingly broad spectrum of processes such as lysosomal biogenesis, autophagy, DNA damage response and innate immunity.^108,109^ In Fig. 5, we summarize the evidence for mTOR activity in squamous epithelia and offer a model: mTOR, especially the mTOR complex 1 (mTORC1) integrates information on the nutritional state of the cell and tissue in regard to glucose, amino acid and oxygen levels and adjusts the behavior of the cells accordingly. If conditions allow growth, mTORC1 inhibits autophagy, lysosome biogenesis and favors protein production by regulating S6K and RPS6, 4EBP1, EIF4E/G and TFE3.

Interestingly, DDIT3, a DDIT4-related mTOR inhibitor protein,^110^ and PML, another mTOR inhibitory protein^111^ and aforementioned DEPTOR are all preferentially expressed in the quiescent basal cell layer of oral mucosa, esophagus, vagina and ectocervix^42^ (Supplementary Fig. S2). These factors also link UPR and hypoxia with mTOR.^112^ PML, the Jack of all trades,^113^ may be an important regulator of mTOR activity by integrating a broad spectrum of stress factors (DNA damage, heat shock, oxidative stress, infection) and affecting transcription and translation.^114^

The question is, which cells are actually utilizing mTOR to control protein translation. The sparse expression data we could gather suggest that basal cells have low levels of mTOR activity (Supplementary Fig. S2, Fig. 5). There is also some evidence of mTOR expression preferentially in suprabasal cell layers rather than basal cells. Collectively, all these data suggest low activity of mTORC1 signaling in the quiescent basal cell layer, which is further supported by our screen of factors that are related to mTORC1 activity, i.e., RPS6, RPS6KB2, EIF4G1, EIF4EBP1, ZKSCAN3 in suprabasal cells and TFE3 (suppressed by mTORC1) in basal cells.

**Fig. 6:**
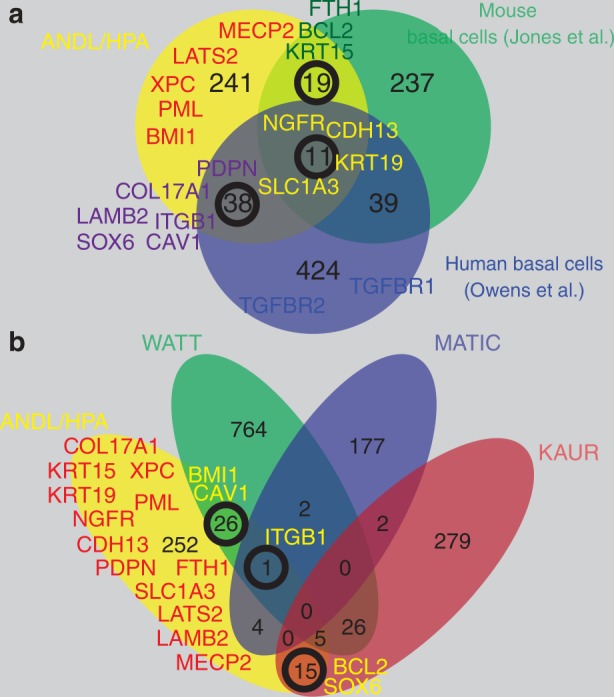
Defining markers of quiescent stem cells. **a** Comparison of three datasets representing analyses of basal cells of squamous epithelia (human esophagus: Owen et al.,^135^ mouse oral mucosa: Jones et al.,^134^ human squamous epithelia ANDL/HPA.)^21^ The majority of genes in each dataset shows no overlap with the other two datasets. **b** Similarly, when comparing datasets on human epidermal stem cells, there is little overlap between publications (WATT;^156,157^ KAUR;^158^ MATIC^159^)

As mTORC1 signaling uses sensors of glucose and amino acid levels as input for its activity, we also interrogated several key transporters and found that surprisingly many of them are preferentially expressed in the basal cell layer. E.g., SLC2A1 (GLUT1) a major glucose transporter is primarily expressed in basal cells^115^ (Supplementary Fig. S2). Amino acid transporters related to mTORC1 signaling show a similar expression pattern (Supplementary Fig. S2): SLC3A2, SLC38A2 (SNAT2, glutamine-leucine anti-porter, import of large neutral amino acids, co-factor of SLC7A5) and SLC7A5 (LAT1, Leucin transporter) are expressed in the basal cell layer. Interestingly, of the 11 genes in Fig. 6a that are identified as stem cell markers in three datasets of oral and esophageal stem/basal cells in mice and humans, two genes are amino acid transporters, SLC1A3 and SLC7A5, and one of them has a critical role in mouse epidermal and hair follicle stem cells.^116^

Much needs to be learned about how mTOR signaling is organized within the oral mucosa and how nutrients flow within the epithelium from the blood supply in the stroma to the suprabasal cells. Despite this limited knowledge, serious and so far promising efforts are underway to use mTOR inhibitors to treat oral cancer.^117^

As all of the key aspects of proteostasis we have explored so far—translational accuracy, translational rate, protein folding appear to be involved in maintaining stemness and quiescence, it is not surprising that the clearance of “bad” proteins also fundamentally contributes to stem cell maintenance. The role of protein clearance systems such as autophagy in stem cell biology is increasingly recognized^118^ and the idea that autophagy could serve as “a quality control mechanism for both proteins and organelles”^118,119^ suggests that some of the most long-lived cells in humans, the tissue stem cells, utilize autophagy to maintain proteostasis as shown in hematopoietic stem cells.^120^ However, little is known on the role of proteostatsis in squamous epithelial stem cell maintenance and activity. Several excellent articles have summarized the current knowledge in other stem cells populations implying a crucial role of the unfolded protein response (UPR), the proteasome, and autophagy in stem cell function and aging and we would like to refer the reader to these resources.^118,121–125^

Collectively, these data on protein translation fidelity and translational control combined with what we know on the effects of diminished DNA repair on premature aging suggest that quality control is an essential thread in the fabric of life on which longevity hinges. Comparisons to manufacturing goods obtrude: the higher the quality of ingredients and machinery to generate a product, the better the chances the product will last a long time. The idea of applying the engineering concept of “mean time to failure” has been around for quite some time.^126,127^

As proteins are main components of cells, it is not surprising that accuracy of the translation of genetic information into protein sequences and the level of protein quality surveillance contribute to stem cell health and longevity.

## Epigenetics of stemness and quiescence

Many aspects of the quiescent reserve stem (qReSt) cells separate them from the other cells of the epithelium. One interesting characteristic is the expression pattern of epigenetic modifiers. The most prominent difference between basal and parabasal cells is in regard to the expression of epigenome modifiers components of the polycomb repressor complexes 1 and 2 (PRC1 and PRC2).^21^ This is particularly interesting as BMI1, a PRC1 component and one of the identifiers of the quiescent stem cells in the intestinal crypts,^128^ is also predominately expressed in the quiescent basal cell layer of the human oral mucosa. In adult mouse HSCs, BMI1 is required to maintain the stem cell population.^129^ Similarly, we have observed that the expression of most PRC1 components (BMI1, RING1, CBX6 [HPA]^21^) are restricted to the basal cell layer, whereas PRC2 members EZH2, SUZ12 and EED are expressed in the proliferating parabasal cells and are low or absent in the basal cell layer.

Mounting evidence suggest that control of the epigenome is key in quiescence. A recent example comes from mouse HSCs, which rely on the epigenome regulator Ash1l to maintain quiescence in adult HSCs.^130^ Without a functional Ash1l adult HSCs cannot maintain their quiescent state and are eventually replaced by progenitor cells to maintain the hematopoietic system. Ash1l-deficient HSCs were not able to function in classical transplantation assays using irradiated mice. Control HSCs restored hematopoietic function in irradiated mice while Ash1l-deficient HSCs failed. However, Ash1l-deficient mice could survive even without quiescent HSCs, indicating that their function is most likely as a quality reservoir for stem cells that also can be used under extreme conditions such as cytotoxic stress or the stressful repopulation of an empty niche in another animal.^130^ Interestingly, the PRC1 component Ring1 mediates gene expression in quiescent B cells and prevents cell death of these resting cells.^131^ Also, in stem cells of acute myelogenous leukemia, Ring1 maintains the stemness of the cancer cells.^132^

In mouse dendritic cells, a polycomb-like protein, Pcgf6, maintains quiescence.^133^ Pcgf6 down-regulation allows dendritic cells to become activated and proliferate. On the other hand, overexpression of Pcgf6 prevented dendritic cell activation.

## The idea of comparative and evolutionary stem cell biology: differences and limitations of mouse stem cell data

Although considerable advances have been made in deciphering the architecture and nature of mouse stem cell populations, normal human adult stem cell populations are still poorly defined. This is worth mentioning as the clear dichotomy of the human undifferentiated cell compartment, characteristic for many human squamous epithelia, is absent in most other mammals including rodents and therefore difficult to explore experimentally.^21^ This raises the question whether findings in our core animal models can actually be translated to the human condition without any key annotations. The uniqueness of a well-defined quiescent cell layer with the presumable ability to regenerate a damaged squamous epithelium after radio-chemotherapy offers to reveal many secrets about human stem cells, longevity, cancer and aging. Therefore, whether the analysis of mouse stem cell populations by themselves will result in an understanding, for example, of human aging is unlikely. However, understanding the differences between humans and animal models may give important clues on how long-living mammals adapt their tissue homeostasis programs to keep their epithelia intact over long periods of time. “Comparative stem cell biology” is still in its infancy as is “evolutionary stem cell biology”. Studying the commonalities and differences in stem cell compartments between different mammalian species may eventually help to answer Peto’s paradox. Large mammals must have developed mechanisms to keep cancer rates low. The nature of these mechanisms is still obscure.

We propose that one potential mechanism to explain Peto’s paradox is to alter the stem cell compartment and modify it in ways to reduce stem cell proliferation. This idea is neither novel nor extraordinary but surprisingly poorly fleshed out. Comparison of stem cell compartments between animals and between tissues seems to offer an option to gain insights how tissue organization and in particular the organization of proliferation within the stem cell compartment may correlate with phenotypes (cancer rates, longevity, response to stress).

Therefore, relying heavily on just data from experimental models based on short-lived rodents may impair progress.

## The great illusion: characterizing the transcriptome of stem cells

The limited usefulness of the mouse as a standalone model for quiescent squamous epithelial stem cells has recently been underscored by a study from Jones et al.^134^ that also included single cell RNA sequencing (scRNA-seq) of mouse oral mucosa cells. This work confirmed that mice do not have label-retaining oral mucosa cells, i.e., quiescent stem cells. An interesting technical aspect of the paper has been the characterization of the epithelium using scRNA-seq. The authors identified two clusters of non-proliferating oral mucosa basal cells distinguished by 302 genes. Only roughly 10% of these genes overlapped with an updated quiescent stem cell marker gene set of human oral mucosa, which we had previously compiled^21^ (Supplementary Table S1; Fig. 6a). We have used the HPA as one of our main sources to identify proteins that are associated with the quiescent basal cell layer. However, one has to consider that the HPA does not cover all coding genes yet, that a substantial number of the antibodies used by the HPA are either not sensitive enough or not specific enough, that RNA analyses are generally more sensitive than protein stainings, and that the RNA data includes non-coding genes that are not considered in the HPA. But even with all these considerations, the overlap between the marker set we have identified and the one from mouse studies, is surprisingly low.

Another scRNA-seq dataset that can help to illuminate the nature of the quiescent stem cell transcriptome stems from Owen et al.^135^ We used this dataset to compare squamous esophageal basal cells (COL17A1^+^ and low in KRT13, RHCG, CRABP2 and TACSTD2) with undifferentiated para-/suprabasal cells (COL17A1^−^ and SPINK5^low^) and identified a set of known and novel markers associated with the basal cell layer of squamous epithelia (Supplementary Fig. S3; Supplementary Table S1). As expected the list includes several integrins, collagens and laminins, as well as NGFR, CDH3, CDH13 and PDPN. TGFbeta, WNT and NOTCH signaling pathway components were enriched. However, a comparison of these data with our quiescent basal stem cell marker list and mouse basal cell markers,^134^ showed little overlap (Fig. 6a).

We observed a similarly low interstudy congruence when comparing different datasets that defined human epidermal stem cells (Fig. 6b and Supplementary Table S1). Three studies evaluating the transcriptome of epidermal stem cells showed only minimal overlap with each other and with our dataset on human squamous epithelial basal cells. The lack of sufficient overlap between the studies is striking and suggests that technical issues and the isolation method of the cells may have a significant impact on the comparability of studies.

As our analyses are based on protein expression and not mRNA, the lack of overlap between our and the other datasets may implicate post-transcriptional mechanisms of gene expression regulation. Multiple antibodies indicate the differential expression of, e.g., MECP2 and XPC, in the basal cell layer of the oral mucosa but there is little evidence on the mRNA level for their differential expression. Future studies have to address the contribution of post-transcriptional events to explain the observed differences between stem cells and their direct descendants. As we have outlined above, there is evidence for differences in translational control in stem cells.

A perplexing aspect of the scRNA-seq data of human esophageal cells is that in the COL17A1+ keratinocytes expression of not just vimentin (VIM) but a set of mesenchymal/fibroblast markers including MCAM, FBLN1, CXCL14, PDPN, CPE, PLAT, SPARC, S100A4 and ACAT2 can be detected; and some of these mRNAs are actually expressed at elevated levels in the COL17A1+ cells when compared to suprabasal cells. On the other hand, there is as expected a complete absence of master regulators of the mesenchymal phenotype such as ZEB1, SNAI1, TWIST1 and TWIST2 from basal cells, whereas SNAI2 mRNA was detected and preferential expressed within the basal cells. In addition to the perplexing expression of mesenchymal marker mRNAs, which is actually not reflected in protein expression data, there is also evidence that the mRNA expression of two markers of simple epithelia and a more undifferentiated status of keratinocytes, i.e., KRT8 and 18, can be detected in basal cells using the Owen et al. dataset. KRT8 is elevated in the basal cells but neither KRT8 nor KRT18 proteins are generally detectable using formalin-fixed, paraffin-embedded tissue material from truly normal squamous epithelia and rather markers of oral malignancy.^136^ Similarly, paradox results were obtained for a subset of interferon-regulated genes and for MHC class II genes. For the MHC class II genes there is no indication of protein expression in keratinocytes, but their mRNAs were present in keratinocyte cells based on our analysis of the Owen et al. data.

Taken together the findings that mesenchymal markers (VIM, SPARC, etc.), MHC class II genes, interferon-regulated genes and markers of simple epithelia (KRT8/18) are detectable on the mRNA level in basal cells of a squamous epithelium are confusing especially as there is little to no evidence that the proteins derived from most of these mRNAs are expressed. Several explanations can be offered. First, some of the genes are expressed at low levels and may represent noise or background. This explanation does not apply for all of these genes as their expression is clearly above such a background and noise level. Second, the expression of these genes could represent an artifact of the procedure to generate single cell suspensions. Some recent studies have explored this issue and found substantial changes upon dissociation of tissue structure and isolation of single cells.^137,138^ This can be summarized with the longstanding warning given by Potten and Loeffler: “One of the major difficulties in considering stem cells is that they are defined in terms of their functional capabilities, which can only be assessed by testing the abilities of the cells, which itself may alter their characteristics during the assay procedure: a situation similar to the uncertainty principle in physics.”^139^ Although there is little evidence to support this kind of artifact in the Owen et al. dataset, we cannot rule out that the processing of the cells introduced changes in their gene expression. E.g., we would expect to see the induction of gene expression associated with keratinocyte activation such as SOX9, MIR31HG or CCL20, but these genes are hardly or not expressed at all in the dataset.^140,141^ A third explanation for this conundrum could be that especially the basal cell layer stem cells exhibit a poised state for several genetic programs associated with keratinocyte activation and epithelial–mesenchymal transition (EMT). This could be tested in the future by exploring the epigenetic state of the genes associated with these programs. Finally, the mRNA expression of these genes that should not be expressed in the basal cells could be regulated on a post-transcriptional level, e.g., by microRNAs. There are hints in the Owen et al. data indicating that microRNAs may contribute at least to some extent to the regulation of mesenchymal mRNAs. E.g., according to our analyses MIR29B2 and MIR99AHG are preferentially expressed in the COL17A1+ cells compared to para- and suprabasal cells. The top predicted target genes in TARGETSCAN^142^ of miR-29 are collagen genes associated with fibroblasts. Furthermore, the epithelial gatekeeper microRNA miR-205HG is expressed in the same cells^143^ and together these microRNAs could in theory prevent the translation of genes associated with EMT.

## Conclusion: the basal cell layer of the human oral mucosa fulfills criteria for a quiescent reserve stem cell pool

In summary, the basal cell layer of the human oral mucosa and other human squamous epithelia is characterized by several factors that appear to be indicators that this cell layer is indeed a reserve stem cell layer:

1. The basal cell layer of these squamous epithelia rarely proliferates and fulfills hallmarks of slow-cycling, undifferentiated stem cells. Accordingly, these basal cells express a TGFbeta regulated gene set indicating that the quiescence may be directly regulated by TGFbeta superfamily members, which are known keratinocyte proliferation inhibitors.
2. These putative qReSt cells are long-lived and label-retaining.
3. These cells reside on the basal lamina, protected by many layers of “dispensable keratinocytes” on top and express genes that are associated with protection from several stress factors.
4. These cells seem metabolically relatively active but may show little translational activity due to their lack of proliferation and therefore the lack of need for protein production. We speculate that most of their energy might be used to maintain gradients or for the transport of nutrients. We further speculate that these cells have low mTORC1 activity.
5. Significant activation of quiescent basal stem cells can be observed under two conditions: irradiation (examples from radio-chemotherapy exposed mucosa shows strong basal cell layer proliferation but hardly any suprabasal cell proliferation), and in pre-malignancy and malignancy the quiescent basal cell layer is abandoned^42^ and discussion therein.
6. Distinct epigenetic gene expression markers especially the separation of polycomb repressor complexes 1 and 2. PRC1 is expressed basally and PRC2 parabasally.
7. Oral mucosa basal cells seem to have low RNA content in line with the general observation that stem cells have low mRNA content.^144–147^
8. Basal cells have longer telomeres than parabasal cells which may indicate that they have less telomere attrition and are less prone to malignant conversion or senescence.^148–150^ Experiments conducted in the oral and esophageal epithelium show that telomerase expression is detected in the basal layer.^151,152^ However, telomeres appear to shorten over lifetime in basal and parabasal cells^150^ and are the shortest in squamous cancer and pre-cancer cells.^129,153,154^ Stress factors that lead to cancer may increase telomere attrition due to higher stem cell proliferation as “normal” epithelia of cancer patients have even shorter telomeres than telomeres in control groups. This may indicate that the carcinogenic environment (tobacco smoke, alcohol) may take a toll on the entire epithelium over the years.

The squamous epithelium of the oral mucosa and its “cousins” in the esophagus, vagina, ectocervix, anus and to some extent the skin is maintained differently in humans than the preferred mammalian model organism, the mouse. The behavior of the stem and progenitor cells appear to be different but their significance for many major pathologies in these epithelia likely is similar. Thus, in our opinion “determining the identity and organization of oral epithelial progenitor cells (OEPCs) is therefore paramount to understanding their roles in homeostasis and disease”,^134^ as Jones et al. have expressed it. However, the current epistemic limitations about the biology of human oral stem cells can only be overcome by acknowledging the differences between species, and by defining the differences with the help of detailed studies of human cells and tissues. It is our opinion that a critical and detailed assessment of studies involving mouse oral biology is not pedantic, but a necessity to make progress in areas such as stem cell biology, aging research, tissue regeneration and cancer research.

## Supplementary information

Expression of heat shock proteins in human oral mucosa

Expression of mTOR signaling related proteins

Expression of basal cell and suprabasal cell markers in human squamous epithelia of oral mucosa and esophagus

Genes associated with human and mouse squamous stem cells and/or basal cells
