# Supplementary figures and images for: Divide and conquer: two stem cell populations in squamous epithelia, reserves and the active duty forces
Source: Int J Oral Sci. 2019 Aug 27;11(3):26. doi: 10.1038/s41368-019-0061-2 (PMC6802623; doi:10.1038/s41368-019-0061-2)

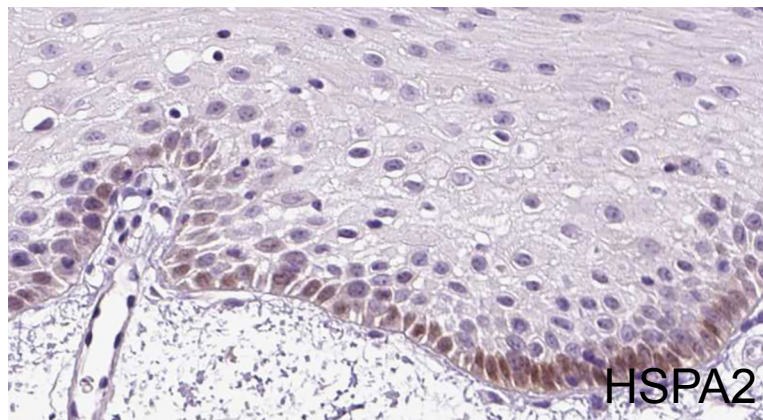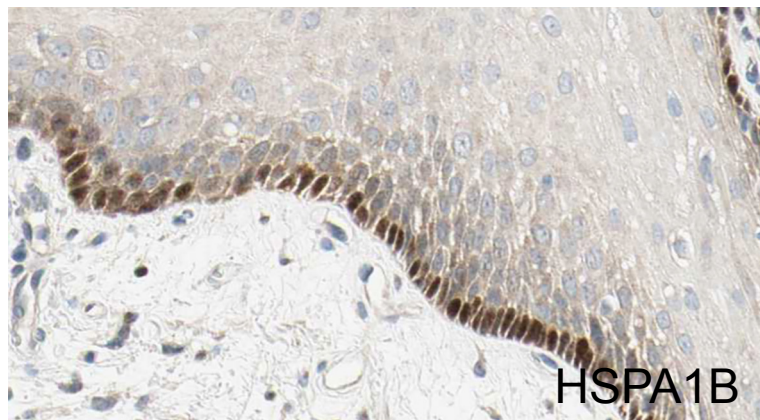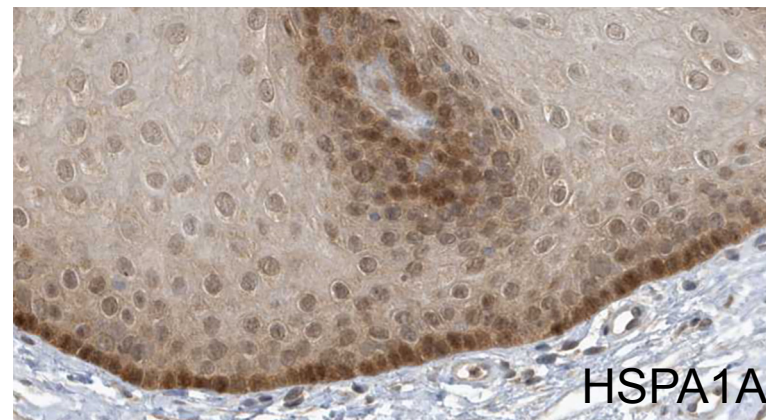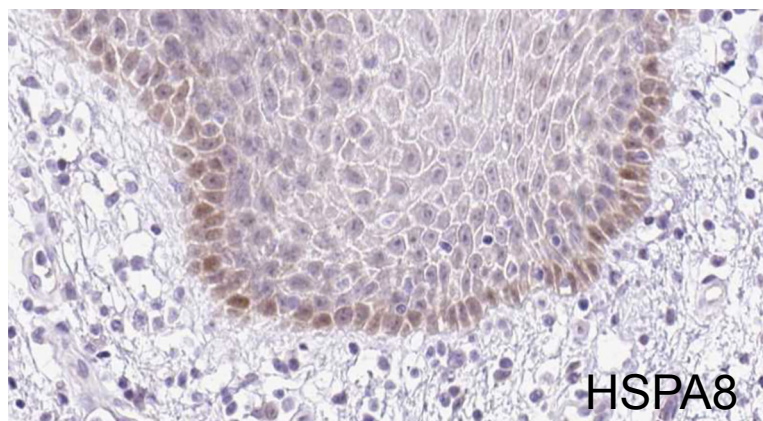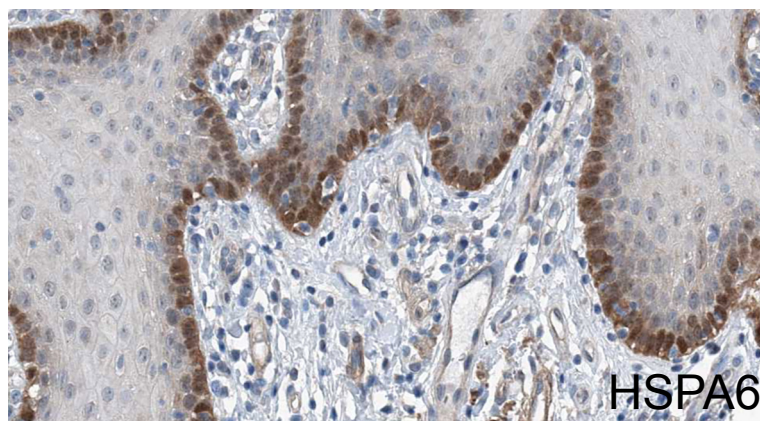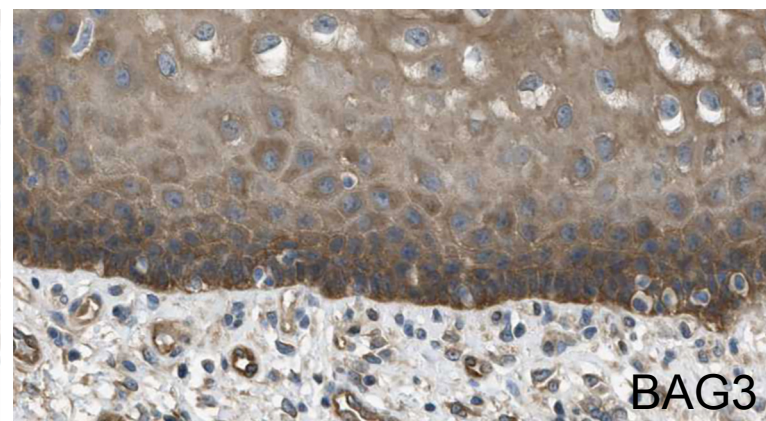

Supplement: Supplementary file 1 — Expression of heat shock proteins in human oral mucosa [file 41368_2019_61_MOESM1_ESM.pdf]

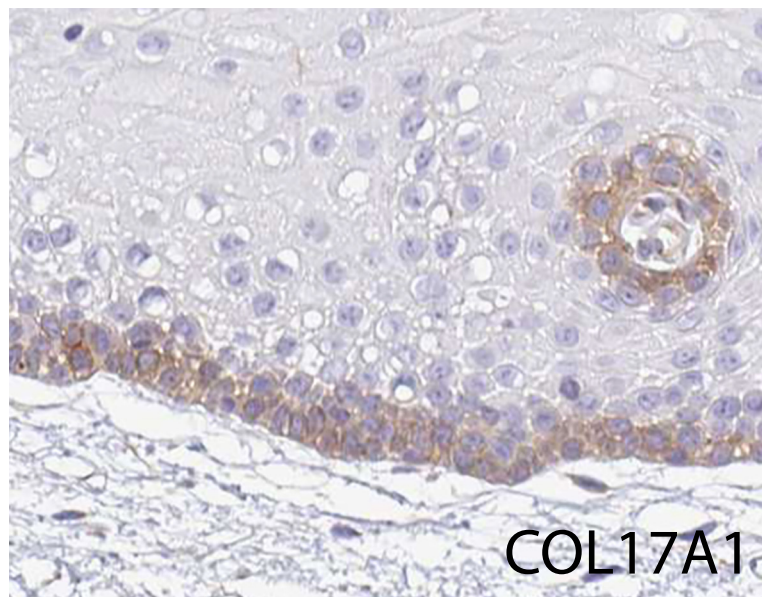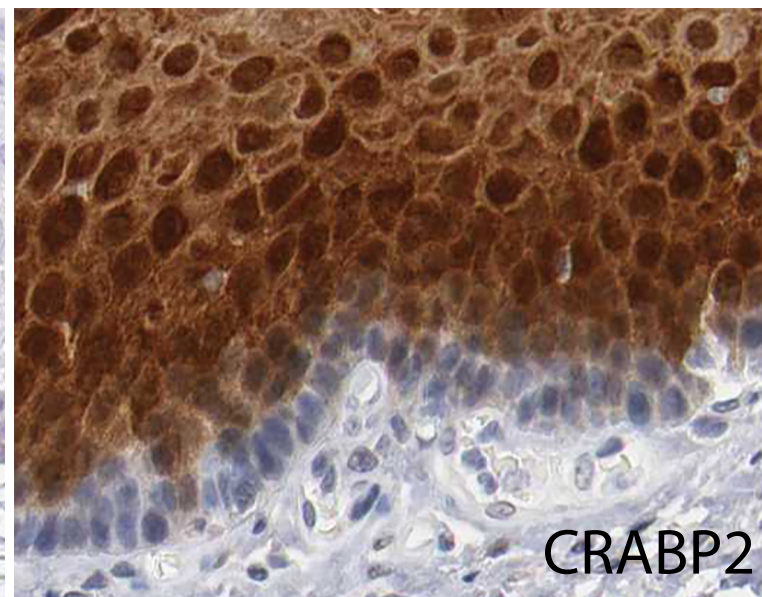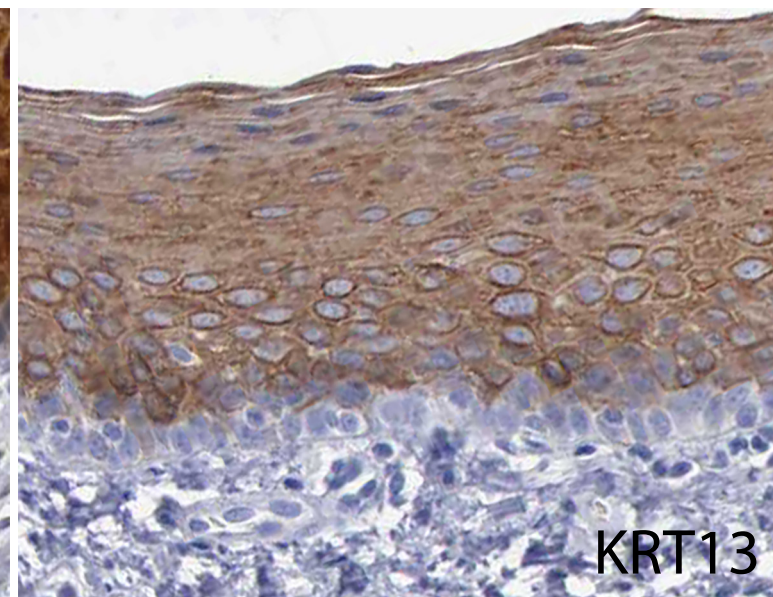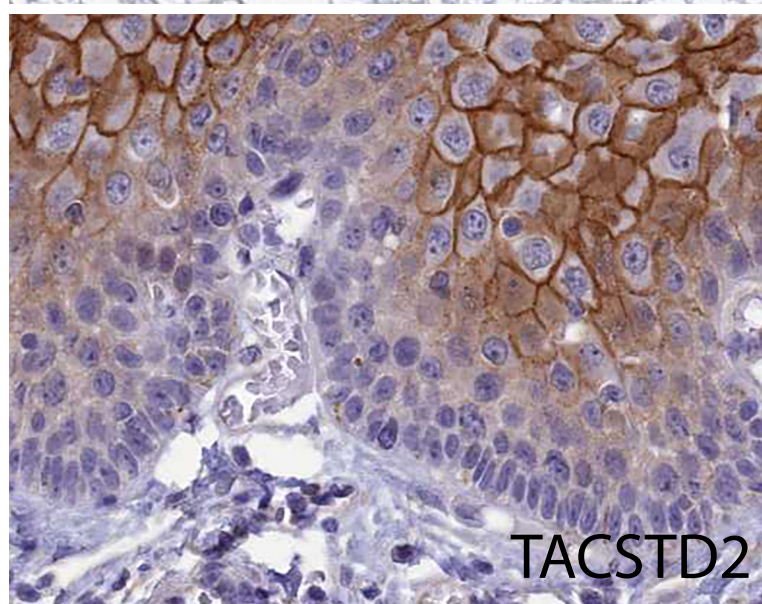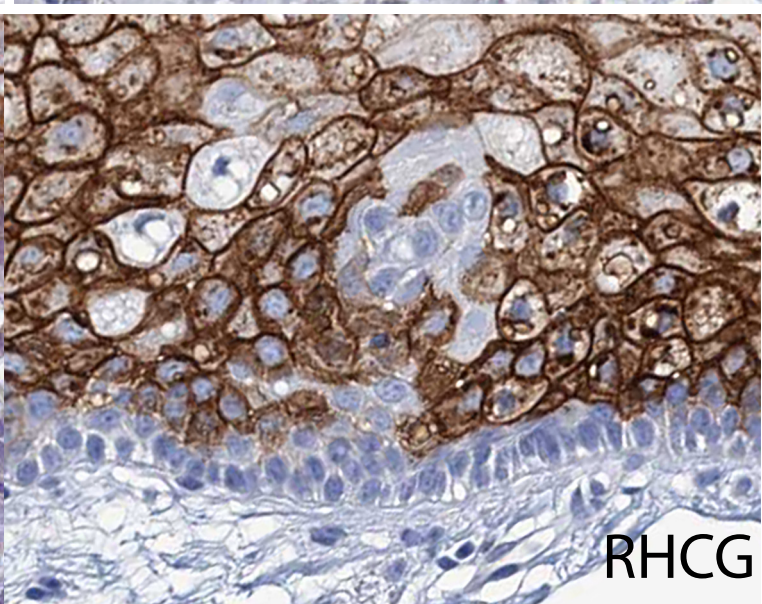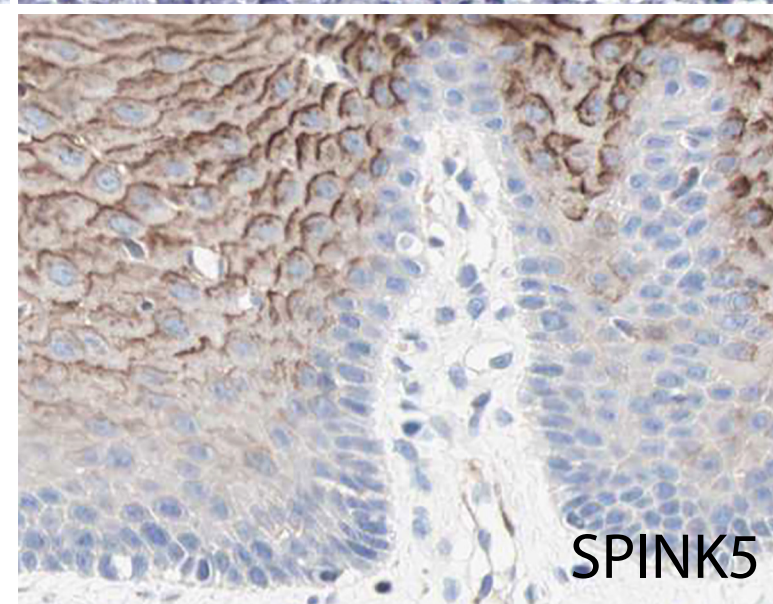

Supplement: Supplementary file 3 — Expression of basal cell and suprabasal cell markers in human squamous epithelia of oral mucosa and esophagus [file 41368_2019_61_MOESM3_ESM.pdf]
