# Supplementary material for: Divide and conquer: two stem cell populations in squamous epithelia, reserves and the active duty forces
Source: Int J Oral Sci. 2019 Aug 27;11(3):26. doi: 10.1038/s41368-019-0061-2 (PMC6802623; doi:10.1038/s41368-019-0061-2)
Supplement: Supplementary file 2 — Expression of mTOR signaling related proteins [file 41368_2019_61_MOESM2_ESM.pdf]

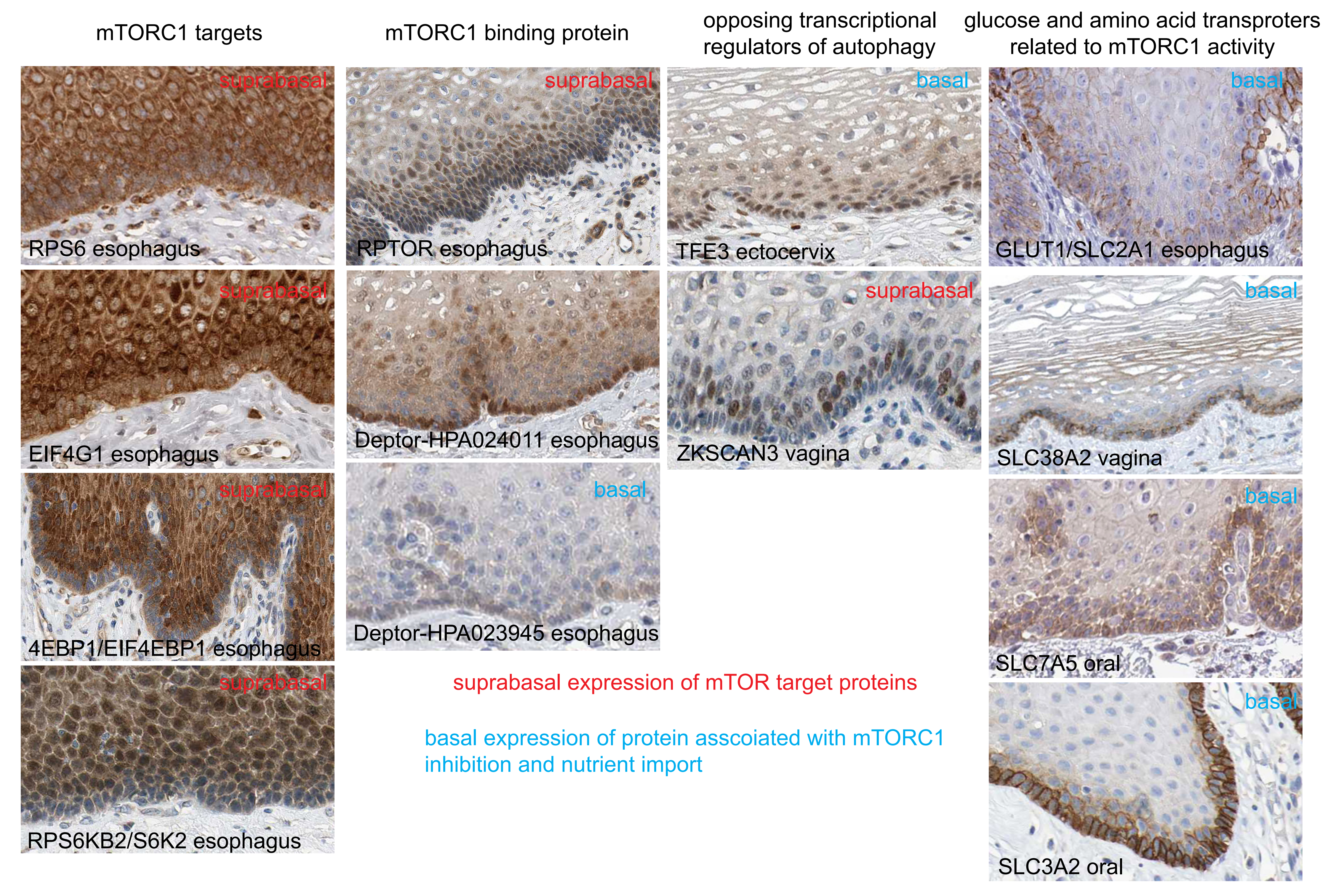
mTORC1 targets
mTORC1 binding protein
opposing transcriptional regulators of autophagy
glucose and amino acid transproters related to mTORC1 activity
suprabasal
RPS6 esophagus
suprabasal
RPTOR esophagus
basal
TFE3 ectocervix
basal
GLUT1/SLC2A1 esophagus
suprabasal
EIF4G1 esophagus
Deptor-HPA024011 esophagus
suprabasal
ZKSCAN3 vagina
basal
SLC38A2 vagina
suprabasal
4EBP1/EIF4EBP1 esophagus
basal
Deptor-HPA023945 esophagus
basal
SLC7A5 oral
suprabasal
RPS6KB2/S6K2 esophagus
basal
SLC3A2 oral
suprabasal expression of mTOR target proteins
basal expression of protein asscoiated with mTORC1 inhibition and nutrient import
